# Assessment of prevalence and risk factors of helicobacter pylori infection in an oilfield Community in Hebei, China

**DOI:** 10.1186/s12876-019-1108-8

**Published:** 2019-11-14

**Authors:** Wenhai Wang, Wei Jiang, Shengtao Zhu, Xiujing Sun, Peng Li, Kejia Liu, Henghui Liu, Junchao Gu, Shutian Zhang

**Affiliations:** 1 Department of Gastroenterology, Beijing Friendship Hospital, Capital Medical University, Beijing, 100050 People’s Republic of China; 2 Beijing Key Laboratory for Precancerous Lesion of Digestive Diseases, Beijing, 100050 China; 3 National Clinical Research Center for Digestive Diseases, Beijing, 100050 China; 4 Beijing Rec data Technology Co, Ltd, Beijing, China; 5 Department of Gastroenterology, Beijing Pinggu Hospital, Capital Medical University, Beijing, 101200 China

**Keywords:** Cross-sectional study, Prevalence, Risk factors, Helicobacter pylori

## Abstract

**Background:**

Only a paucity of large-scale perspective and cross-sectional studies on *H. pylori* infection in China have been published. The purpose of this study was to investigate the prevalence and risk factors for *H. pylori* infection among residents of Jidong community located in Hebei Province of China.

**Methods:**

A perspective, cross-sectional study was conducted in Jidong community. Questionnaires and ^13^C-urea breath test were performed, and 10-ml blood samples were obtained for laboratory tests.

**Results:**

Four thousand seven hundred ninety-six subjects were enrolled in this study, and 2506 (52.25%) were *H. pylori* positive. There was no difference in prevalence between both sexes (*P* = 0.5974). Age (*P* = 0.004) and education level (*P* = 0.0128) were significantly associated with *H. pylori* infection, and there were statistical trends in the prevalence across five age subgroups (χ^2^ test for trend = 23.5; *P* < 0.001) and education levels (χ^2^ test for trend = 19.50; *P* < 0.001). *H. pylori* infection was also associated with marital status (*P* = 0.0243), source of drinking water (*P* = 0.0433), frequency of eating raw garlic (*P* = 0.0310), alcohol drinking (*P* = 0.0207), knowledge about *H. pylori* transmission route (*P* = 0.0125) and related diseases (*P* = 0.0257). Age, alcohol drinking and knowledge about transmission route were found to be independent predictors of *H. pylori* infection.

**Conclusions:**

More than half of the population was infected with *H. pylori* in Jidong community. The socio-demographic profiles, socio-economic factors and lifestyle are worthy taking into consideration to prevent diseases associated with *H. pylori* infection. Understanding the prevalence and risk patterns for *H. pylori* infection in China will help in prioritizing public health efforts to better manage the *H. pylori* infection.

## Background

Firstly discovered by Warren and Marshall in 1983 [1], Helicobacter pylori (*H. pylori*) has coexisted with humans for a relatively long period and has been widely recognized to be a potent pathogenic factor that may cause gastritis, peptic ulcers, gastric mucosa-associated lymphoid tissue lymphoma, and gastric cancer [2]. Lately, its involvement in other diseases such as abnormalities in autoimmune systems, cardiovascular diseases, and metabolic syndrome has attracted a great deal of attention of researchers and physicians [3].

To date, nearly 50% of the global population has been in infectious status with this bacterium [4]. The incidence of *H. pylori* infection can be affected by hygienic conditions and socioeconomic status. Taking it as an instance, the incidence of *H. pylori* infection in UK and USA was 13.4 and 27.1%, respectively [5] while in undeveloped nations such as Chile, Turkey and Bangladesh, its prevalence can reach 73.4, 74.6 and 92% [6–8]. The disparity of infection rate has been described in a great deal of studies, however, the exact route of transmission and potential risk factors have not been well elucidated.

Currently, the *H. pylori* infection has posed a serious challenge to public health in China. Even though accumulating evidence has illustrated the incidence of *H. pylori* in many regions [9–14] (Table 1), few large-scale cross-sectional studies recruiting asymptomatic subjects have been carried out in average-risk community. Jidong community is a relatively enclosed area in Hebei Province. Local residents are mainly composed of employees working for the Jidong Oilfield Inc. and their family members. Within this community, there is considerable homogeneity concerning the high socio-economic levels, gradual population shifts and limited communication with the people outside of this community.

**Table 1:** Summary of previous Helicobacter pylori prevalence studies in China

Author	Study Period	Setting	Area	Age group	Number (n, M/F)	Prevalence	Prevalence (%, M/F)	Test
Wang	1989	General population	69 counties	35–64	8280 (only men)	71.4	N/A	ELISA IgG-Ab
Zhu	09–11	Healthy individuals	Eastern China	30–69	5417 (2342/3075)	63.41	61.74/64.47	^13^C-UBT
Shi	04–05	Healthy individuals	Eastern China	5–100	1371 (585/786)	62.07	61.96/62.07	^13^C-UBT
Yu	07–11	Children with GI discomfort	Eastern China	≤18	1634 (865/769)	32.1%	N/A	Histology, Urease test
Chen	2003	Healthy individuals	southern China	3–92	1471 (760/711)	47%	47.1/47	ELISA IgG-Ab
Zhang	2010	Aged people	Northern China	≥60	2006 (1005/1001)	83.4%	84.7/82.1	ELISA IgG-Ab

To examine the prevalence and risk factors for *H. pylori* infection in Jidong community, we designed a large-scale, prospective, cross-sectional study named Helicobacter pylori Infection in Oilfield Community (HIOC) Study, which described the results of the cross-sectional investigation on prevalence and risk factors for *H. pylori* infection in Jidong community.

## Methods

### Research and design of the study population

This was a cross-sectional study carried out in Jidong Community of Hebei province, China. This community included 10,043 employees of the Jidong Oilfield Inc. and their families. From June 2016 to June 2017, all residents took physical examination in the Caofeidian District Hospital. Six thousand six hundred fifty-six subjects agreed to participate this study and completed a structured questionnaire. One thousand eight hundred sixty subjects were excluded from the study. The exclusion criteria were as follows: 1. Past history of gastric cancer, coronary heart disease (CHD) or ischemic stroke (IS) as assessed by a validated questionnaire; 2. Previous gastrectomy; 3. On antibiotics during past 30 days or any proton pump inhibitors within 14 days before. Finally, a total of 4796 consecutive subjects were enrolled and subsequently had ^13^C-urea breath test (13C-UBT).

The study protocol was reviewed and approved by the Ethics Committee of Jidong Oilfield Inc. Medical Center and Beijing Friendship Hospital, Capital Medical University. The approval will be renewed every 5 years. We got written informed consent from all participants.

### Clinical and laboratory evaluations

The data such as weight, height, waist and hip circumference of all participants were recorded on their first visits to Caofeidian District Hospital. A 10-ml blood sample of each subject was obtained from the antecubital vein in the morning after overnight fasting, and serum samples were separated after centrifugation and were stored in − 70 °C until analysis. Serum cholesterol, triglyceride, and fasting glucose were measured by an autoanalyzer (Olympus, AU400, Japan) at the central laboratory in the Caofeidian District Hospital. To compare these results according to prevalence of *H. pylori* infection, we categorized the level of total cholesterol (TC) as usual (<5.2 mmol/l) and abnormal (≥5.2 mmol/l), triglyceride (TG) as standard (<1.7 mmol/l) and irregular (≥1.7 mmol/l), and fasting glucose as normal (<6.1 mmol/l) and abnormal (≥6.1 mmol/l), respectively.

### Questionnaires

All subjects were invited to finish a structured questionnaire under the guidance of a well-trained interviewer (Additional file 1). The questionnaire captured the information regarding demographic and socioeconomic characteristics (i.e., age, sex, marital status, BMI, TC, TG, and fasting glucose, education level, source of drinking water, household population, Household hygiene, household surrounding environment and household area per capita), personal habits and knowledge about *H. pylori* (i.e. cigarette smoking, alcohol drinking, intake of raw garlic, raw vegetables, fried foods and pickled foods, hand washing before meals and after toilet use, staying up at night, peeling fruits, raising domestic animals, sharing cutlery or cups, eating by individual serving), and medical history (i.e. chronic gastritis, gastric ulcer, duodenal ulcer, chronic cholecystitis and cholelithiasis, previous gastroscopy, family history of GI cancer, hypertension, diabetes mellitus, hyperlipidemia). Body mass index (BMI) was classified into 3 grades: normal status (≤25 kg/m2), overweight (25 kg/m^2^ to 30 kg/m^2^), and obesity (≥30 kg/m^2^) [8]. We defined the condition of household hygiene as good if the participant or his/her families can clean their house once per day, as intermediate if per week, as bad if more than 1 week. Household surrounding environment was referred to as good if public trash cans can be cleared more than twice per day, as intermediate if once per day, as bad if only cleared once every 2 days or even longer.

### Breath sample collection

After obtaining informed consent from participants the status of *H. pylori* infection would be assessed using ^13^C-UBT which was undertaken after at least 6 h of fasting. The ^13^C-UBT was performed using 75 mg of ^13^C-urea dissolved in 100 mL of drinkable water. Breath samples were collected before and 30 min after drinking water. Then they were analyzed using a nondispersive infrared spectrometer (HY-IREXB, Hua You Scientific, China). We regarded the result as positive when the value was equal to or higher than 4 ± 0.4. Certain subjects with positive results would be advised to eradicate *H. pylori*.

### Statistical analyses

The baseline characteristics of participants with or without *H. pylori* infection were compared, using the chi-square test for categorical variables and the two-sample t-test for continuous variables. The trend of *H. pylori* infection rate in different age groups was analyzed by chi-square trend test. A *P*-value of <0.05 was considered to be statistically significant. Odds ratios (OR) and 95% confidence intervals (CI) for the association between *H. pylori* infection and potential risk factors were estimated using multivariable logistic regression models. Variables were selected for entry into the regression model if the variables were significantly associated with *H. pylori* infection (*p* < 0.1) in the univariate analysis. All analyses were performed using SAS 9.3 (SAS Institute, Cary, North Carolina, USA).

## Results

### Demographic, socioeconomic characteristics and *H. pylori* infection

The demographic features of 4796 subjects were summarized in Table 2. The mean age was 44.46 ± 13.45 years old, with 49.9%(*n* = 2392) being male (male-to-female ratio of 1:1.01). The majority of participants were currently married (4140/4796, 86.3%). More than half of the respondents had a high education level (university or above, 57.8%). The majority of them used barreled water as a main source of drinking water (56.0%). Nearly half of the population had household area per capita of 30–60 m^2^ (44.2%) and the household population of middle size, good condition of household hygiene, good surrounding environment accounted for 90.1, 67.9 and 66.0%, respectively.

**Table 2:** Description of socio-demographic and socioeconomic characteristics

Details	Frequency n (%)
Socio-demographic characteristics	
Age (*n* = 4796)	
19–30	858 (17.9)
31–40	1293 (27.0)
41–50	1001 (20.9)
51–60	797 (16.6)
> 60	847 (17.7)
Sex (*n* = 4796)	
Male	2392 (49.9)
Female	2404 (50.1)
Marital status (*n* = 4796)	
Currently married	4140 (86.3)
Single	656 (13.7)
Socioeconomic characteristics (*n* = 4435)	
Education level	
Primary	296 (6.7)
Middle or high school	1575 (35.5)
University or above	2564 (57.8)
Household area per capita (*n* = 4793)	
≤ 30 m2	1196 (25.0)
30–60 m2	2118 (44.2)
≥ 60 m2	1479 (30.9)
Household population (*n* = 4794)	
1	163 (3.4)
2–5	4317 (90.1)
≥ 5	314 (6.5)
Household hygiene (*n* = 4794)	
Good	3256 (67.9)
intermediate	1517 (31.6)
Bad	21 (0.4)
Household surrounding environment (*n* = 4793)	
Good	3163 (66.0)
intermediate	1593 (33.2)
Bad	37 (0.8)

Minority of respondents reported a medical history of chronic gastritis (11.0%), gastric ulcer (3.6%), duodenal ulcer (2.2%), chronic cholecystitis and cholelithiasis (5.7%), hypertension (28.9%) and diabetes mellitus (9.0%), but nearly half of them reported hyperlipidemia (48.2%). Only 10.0% of the respondents had the previous gastroscopy, and 1.3% among respondents said a family history of gastrointestinal cancers (Table 3). Univariate analysis showed no significant differences across medical histories. (Ps > 0.5).

**Table 3:** Description of medical history

Details	Frequency n (%)
Chronic gastritis (*n* = 4794)	
No	4267 (89.0)
Yes	527 (11.0)
Gastric ulcer (*n* = 4793)	
No	4622 (96.4)
Yes	171 (3.6)
Duodenal ulcer (*n* = 4793)	
No	4687 (97.8)
Yes	106 (2.2)
Chronic cholecystitis or cholelithiasis (*n* = 4794)	
No	4522 (94.3)
Yes	272 (5.7)
Previous gastroscopy (*n* = 4793)	
No	4313 (90.0)
Yes	480 (10.0)
Family history of GI cancer (*n* = 4792)	
No	4731 (98.7)
Yes	64 (1.3)
Hypertension (*n* = 4796)	
No	3409 (71.1)
Yes	1387 (28.9)
Diabetes mellitus (*n* = 4796)	
No	4365 (91.0)
Yes	431 (9.0)
Hyperlipidemia (*n* = 4796)	
No	2485 (51.8)
Yes	2311 (48.2)

### The prevalence of *H. pylori* infection

**Fig. 1:**
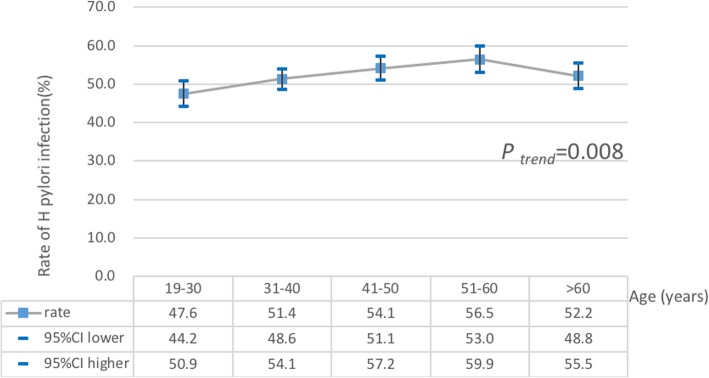
Age-specific prevalence of Helicobacter pylori infection diagnosed by ^a 13^C-urea breath test. There was a marked difference between age groups (*P* = 0.0045) and an increasing trend of prevalence with age (P trend = 0.008)

The overall prevalence of *H. pylori* was 52.25% (95% CI = 50.84–53.67%) in terms of the results of 13C-UBT from all subjects. The age-specific prevalence of *H. pylori* infection diagnosed by ^13^C -UBT is shown in Fig. 1 It illustrated a marked difference between age groups (*P* = 0.0045) and an increasing trend of prevalence with age (χ^2^ test for trend = 23.5; *P* < 0.001).

It is depicted in Table 4 that there was no significant difference between both sexes (*P* = 0.5974) while different marital status showed significant difference in prevalence (*P* = 0.0243). Subjects who were currently married were more prone to be infected than those who were single. The prevalence of *H. pylori* was lowest (50.4%) when the educational level of the patient was highest, and there was a negative association between education level and *H. pylori*’s infection in general (χ^2^ test for trend = 19.50; *P* < 0.001). Concerning the source of drinking water, subjects who used piped water were at higher risk for *H. pylori* infection than those who used barreled or bottled water (53.9% vs. 50.9%, *P* = 0.0433).

**Table 4:** Relationships between the prevalence of *H. pylori* infection and risk factors

	Hp positive		Hp negative			
	n	(%)	n	(%)	Total	P
Sex						
Male	1259	52.6	1133	47.4	2392	0.5974
Female	1247	51.9	1157	48.1	2404	
Marriage status						
Married	2190	52.9	1950	47.1	4140	0.0243
Single	316	48.2	340	51.8	656	
Education level						
Primary school	162	54.2	134	45.8	296	0.0128
Middle/high school	871	55.3	704	44.7	1575	
University/above	1292	50.4	1272	49.6	2564	
No data	181	50.1	180	49.9	361	
Source of drinking water						
Piped water	1135	53.9	972	46.1	2107	0.0433
Barreled water	1368	50.9	1318	49.1	2686	
Frequency of eating garlic						
Never/seldom	1974	51.5	1861	48.5	3835	0.0310
Often/everyday	532	55.4	429	44.6	961	
Alcohol drinking						
Yes	832	54.7	689	45.3	1521	0.0207
No	1674	51.1	1601	48.9	3275	
Cigarette smoking						
Yes	565	53.6	490	46.4	1055	0.3375
No	1941	51.9	1800	48.1	3741	
Route of transmission						
Unclear	2351	52.7	2106	47.3	4457	0.0125
Clear	154	45.7	183	54.3	337	
*H. pylori*-related diseases						
Unclear	2362	52.7	2122	47.3	4484	0.0257
Clear	144	46.2	168	53.8	312	

### Anthropometric values and prevalence of *H. pylori* infection

The proportion of the participants with normal anthropometric values included BMI < 25 kg/m^2^ (60.5%), TC < 5.18 mmol/L (63.7%), TG < 1.7 mmol/L (59.8%) and Fasting Glucose<7.0 mmol/L (89.1%). For *H. pylori* positive and negative subjects, the mean value of BMI, TC, TG and fasting glucose were 24.59 ± 3.59 and 24.48 ± 3.67, 4.94 ± 0.96 mmol/L and 4.91 ± 0.94 mmol/L, 1.91 ± 1.61 mmol/L and 1.81 ± 1.34 mmol/L, 6.02 ± 1.33 mmol/L and 5.99 ± 1.30 mmol/L, respectively. No significant differences were obtained between subjects with and without *H. pylori*(Ps > 0.5).

### Association between *H. pylori* positivity with personal habits and knowledge about *H. pylori*

No significant difference was obtained between smokers and non-smokers for *H. pylori* prevalence (*P* = 0.3375). However, patients who consumed alcohol had a higher prevalence of active *H. pylori* infection compared to non-drinkers (*P* = 0.0207). Subjects who never or seldom ate raw garlic had a lower *H. pylori* infection rate than those who ate often or every day (51.5% vs. 55.4%, *P* = 0.0310) **(**Table 4**)**. Respondents who were familiar with the route of transmission and related diseases of *H. pylori* had a lower prevalence of infection than those who were unaware of (*P* = 0.0125, *P* = 0.0257, respectively).

### Logistic regression model analysis for *H. pylori* infection

Multivariate logistic regression analysis revealed that following factors were significant independent variables: age (*P* = 0.0024), alcohol drinking (OR = 1.139, 95% CI = 1.025–1.290, *P* = 0.0407) and knowledge about the route of transmission (OR = 0.796, 95% CI = 0.635–0.996, *P* = 0.0463). Respondents aged 41–50 (OR = 1.308, 95% CI = 1.088–1.573, *P* = 0.0043), 51–60 (OR = 1.460, 95% CI = 1.200–1.777, *P* = 0.0002) and over 60 years old (OR = 1.239, 95% CI = 1.021–1.502, *P* = 0.0296) were more likely to be *H. pylori* positive compared to those of 30 years old and below (Table 5).

**Table 5:** Multivariate logistic regression analysis of *H. pylori* infection

Variables and categories	OR (95%CI)	P
Age		0.0024
19–30	Reference	
31–40	1.154 (0.970–1.374)	0.1053
41–50	1.308 (1.088–1.573)	0.0043
51–60	1.460 (1.200–1.777)	0.0002
>60	1.239 (1.021–1.502)	0.0296
Alcohol drinking	1.139 (1.005–1.290)	0.0407
Knowledge about route of transmission	0.796 (0.635–0.996)	0.0463

## Discussion

This was the first large-scale cross-sectional study which estimated the prevalence and potential risk factors for H pylori infection by using ^13^C-UBT in an average-risk of gastric cancer region in China. In our study, the overall prevalence of *H. pylori* infection was 52.26%. It was associated with age, marital status, education level, source of drinking water, frequency of eating garlic, alcohol drinking, knowledge about the route of transmission and *H. pylori*-related diseases. Age, alcohol drinking, and experience about the course of transmission were independent indicators for H pylori infection.

The prevalence of the *H. pylori* infection in this study (52.26%) was in consistence with an average *H. pylori* IgG seropositive rate of 58.07% in a meta-analysis of five cohort studies, 18 case-control studies and 66 cross-sectional studies published from 1990 to 2002 in China [9]. As to the potential risk factors for *H. pylori* infection such as demographic characteristics, anthropometric measurements, socioeconomic status, and personal habits, currently the results are still controversial.

Darko et al. [15] and Naja et al. [16] reported that the infection of *H. pylori* was closely associated with gender distribution. However, there was no significant correlation in our study. Multivariate logistic regression analysis of previous studies revealed that age was a potent risk factor for *H. pylori* infection and many studies have reported that the prevalence of *H. pylori* infection increased by age manner [17–19]. Similarly, we found the prevalence of *H. pylori* infection increased with age, peaking at the age group of 51–60. In the multivariate analysis, age was also found to be a significant predictor for *H. pylori* infection.

The *H. pylori* can usually transmit in direct and indirect manners. Direct transmission involves the intimate interaction, while indirect transfer requires vehicles like air, drinking water, food, flies, or other animals [20]. For example, it has been reported that an open mouth kiss or sharing cutlery/cups may transmit *H. pylori* by an oral-oral route. Brenner et al. [21] observed that the risk for *H. pylori* infection would get higher as the time living with the spouse with *H. pylori* infection went by. In our study, we found the similar result that currently married couples bore a higher risk for *H. pylori* infection than others who were single. The waterborne route is also a well-known manner of *H. pylori* infection since studies from different nations found a strong correlation between *H. pylori* infection and the source of drinking water [22, 23]. In our study, participants who drunk piped water were at higher risk than those who used barreled water as drinking source which was in line with what previous studies have reported.

*H. pylori* infection was also associated with food and eating habits [24]. Previous studies have reported that the garlic can have protective effect against Helicobacter pylori [25] and a positive correlation can be observed between eating kipper/fried food and *H. pylori* infection [10]. Interestingly, we found a higher rate of H pylori infection was denoted with the increasing amount of raw garlic consumption, which was contrary to most previous studies. The exact reason still needs to be further investigated.

Several previous studies have investigated the relationship between *H. pylori* infection and alcohol or cigarette consumption, but no agreement has reached yet. Two studies from China reported that there was no significant correlation between *H. pylori* infection and smoking or drinking [10, 11]. However, one study from Japan reported that smoking is inversely associated with *H. pylori* infection [26]. We found that there was no significant correlation between smoking and *H. pylori* infection. Furthermore, it has nothing to do with the quantity of cigarettes consumed per day. The prevalence of *H. pylori* infection has been previously reported to be negatively associated with the amount of alcohol consumption [27]. Similarly, Murray et al. found that alcoholic beverages has such effect by facilitating the eradication of *H. pylori* [28]. The mechanism of alcoholic beverages eradicating *H. pylori* might be the increased gastric acid secretion and gastric emptying once consumed [29]. Furthermore, it was plausible that alcoholic drinks had vigorous, direct antibacterial activity as investigated in vitro [30]. Different from previous studies [24, 31], we found that consumption of alcohol was positively correlated with *H. pylori* infection and was considered as an independent risk factor for *H. pylori* infection by multivariate regression analysis. The reason is probably due to heterogeneity and the disparity in the quantity of alcohol consumption between different populations.

Since *H. pylori* infection was defined as an infectious disease, a number of education programs have been carried out to advance the detection and management for such infection in public. One survey recruiting outpatients in China revealed that 60% of patients only knew two items from total seven questions about *H. pylori* in questionnaires, while 46.7% patients wanted to obtain the knowledge of the disease and instructions on health care from medical professionals [27]. In this study, we found that a majority of participants had poor experience about *H. pylori*, and participants who were familiar with the route of transmission and *H. pylori*-related diseases had a lower prevalence of infection. Particularly, knowledge about *H. pylori* transmission can be regarded as an independent protective factor in multivariate analysis.

However, there were several limitations in our study. First, the relationship between *H. pylori* infection and corresponding risk factors in this study could not be confirmed completely, which is probably due to the design of the cross-sectional study. Further explanations of these results would be addressed during prospective follow-up period. Second, although the inhabitants of Jidong community came from different parts of China, the results of this study still might not be a good representation of the general population. Moreover, evidence derived from any cross-sectional study is generally considered of more inferior methodological quality than randomized trials. Thus, despite our delicate study design and deliberate attempts to control confounding variables, bias could remain due to unmeasured or unknown confounders.

## Conclusion

The overall prevalence of *H. pylori* infection was 52.26% in the populations of Jidong community. The prevalence of *H. pylori* infection was associated with age, marital status, education level, source of drinking water, frequency of eating garlic, alcohol drinking, knowledge about the route of transmission and *H. pylori*-related diseases. Multivariate logistic models noted that age, alcohol drinking, and experience about the course of transmission were independent indicators for H pylori infection. The relationship between *H. pylori* infection and several factors such as personal habits and types of food consumed needs to be further investigated.

## Supplementary information

**Additional file 1. **Description of the questionnaire used for health follow-up in Jidong Community. The data from this questionnaire includes information about socio-demographic and socioeconomic characteristics, personal habits, medical history and knowledge about *H. pylori* of residents in Jidong Community.

## Data Availability

All data generated or analyzed during this study are included in this published article [and its supplementary information files].

## Abbreviations

BMI: Body mass index
CI: Confidence interval
C-UBT: C-urea breath test
*H. pylori*: Helicobacter pylori
OR: Odds ratio
TC: Total cholesterol
TG: Triglyceride
