# Supplementary material for: Assessment of prevalence and risk factors of helicobacter pylori infection in an oilfield Community in Hebei, China
Source: BMC Gastroenterol. 2019 Nov 14;19:186. doi: 10.1186/s12876-019-1108-8 (PMC6857281; doi:10.1186/s12876-019-1108-8)
Supplement: Supplementary file 1 — Additional file 1. Description of the questionnaire used for health follow-up in Jidong Community. The data from this questionnaire includes information about socio-demographic and socioeconomic characteristics, personal habits, medical history and knowledge about H. pylori of residents in Jidong Community. [file 12876_2019_1108_MOESM1_ESM.pdf]

DECLARATION

THE FOLLOWING IS THE ENGLISH VERSION OF THE QUESTIONNAIRE REFERRED IN THIS MANUSCRIPT. IT'S ONLY PART OF THE WHOLE QUESTIONNAIRE BECAUSE THE UNINVOLVED PART IS ASSOCIATED WITH OTHER PROJECTS. ANYWAY, THE CONTENT IS RELATIVELY COMPLETE IN TERMS OF THE DATA WE REFERRED IN THIS MANUSCRIPT.

IN THE EVENT OF INCONSISTENCY OR DISCREPANCY BETWEEN THE CHINESE VERSION AND THE ENGLISH VERSION, THE CHINESE L ANGUAGE VERSION SHALL PREVAIL.

Corresponding author: Shutian Zhang

Signature: ______________

Health follow-up form in Jidong Community

File number: ____________________

Health file number: ____________________

Name of the participant: ____________________

Department: ____________________

Date: ____________________

Name of the investigator: ____________________

Version of 2015

File number: ____________

Informed Consent

I have read and understood adequately the procedure, which the doctor and investigator involved in this study explained to me.

I hereby consent to undergo the examination. And I am willing to undertake the potential risk and detrimental results of the procedure.

DORTOR'S DECLARATION: I have fully explained the nature, risks and benefits of the study to the above patient/relative/guardian, and answered their questions. To the best of my knowledge, the patient/relative/guardian has been informed adequately and has consented. The medical staff will be responsible for the explanation of the examination results.

IN THE EVENT OF INCONSISTENCY OR DISCREPANCY BETWEEN THE CHINESE VERSION AND THE ENGLISH VERSION, THE CHINESE LANG UAGE VERSION SHALL PREVAIL.

Participant:

signature:___________ or relative/guardian signature:_________ Relationship:_________

Doctor signature: _______________ Institution: _______________

Investigator signature: ______________ Institution: _______________

File number: ___________

Part one: Basic Information

A1. Name: ___________ A2. Gender: ___________ A3. Neck Circumference: __________cm

A4. Phone (Mobile): _______________

A5. Phone (immobile): _______________

A6. ID Number: ______________________________

A7. Address：______ Proveince _______ City_______ County________Village

A8. Household area per capita（sq.m）: <30 _______ 30-60 _______ >60 _______

A9. Household Population: 1_______ 2-4_______ ≥5_______

A10. Surrounding Environment: Good_______ Intermediate_______ Bad________

A11. Household Hygiene: Good_______ Intermediate_______ Bad________

A12. Education Level: Primary School_______ Middle or high school:_____ University or above:________

A13. Marriage Status: Single: _______ Married: _______ Divorced: _______ Other: _______

File number: ____________

Part two: Disease Information

1. History of Cardiovascular diseases

_____Atrial Flutter/Atrial Fibrillation Date: ___________ Hospital:____________

_____Heart Failure Date: ___________ Hospital:____________

_____Myocardial Infarction Date: ___________ Hospital: ____________

_____Coronary Heart Disease Date: ___________ Hospital: ____________

_____Peripheral Artery Atherosclerosis Date: ___________ Hospital: ___________

_____Coronary Stent Implantation Date: ___________ Hospital: ___________

2. History of Cerebrovascular diseases

_____Ischemic Stroke Date: ___________ Hospital:____________

_____Cerebral Hemorrhage Date: ___________ Hospital:____________

_____Subarachnoid Hemorrhage Date: ___________ Hospital: ____________

_____Transient Ischemic Attack Date: ___________ Hospital: ____________

_____Others Date: ___________ Hospital: ___________

3. History of Cancer

_____Breast Cancer Date: ___________ Hospital:____________

_____Lung Cancer Date: ___________ Hospital:____________

File number: ____________

_____Gastric Cancer Date: ___________ Hospital: ____________

_____Colorectal Cancer Date: ___________ Hospital: ____________

_____Pancreatic Cancer Date: ___________ Hospital: ___________

_____Renal Cancer Date: ___________ Hospital: ___________

_____Ureter Cancer Date: ___________ Hospital: ___________

_____Bladder Cancer Date: ___________ Hospital: ___________

_____Prostatic Cancer Date: ___________ Hospital: ___________

_____Thyroid Cancer Date: ___________ Hospital: ___________

_____Lymphoma Date: ___________ Hospital: ___________

_____Leukemia Date: ___________ Hospital: ___________

_____Cervical Cancer Date: ___________ Hospital: ___________

_____Endometrial Cancer Date: __________Hospital: ___________

_____Others Date: _____________ Hospital: ___________

Part two: Personal Habits

1. History of smoking

1.1 Smoking Yes. ___________ No. ___________

1.2 Amount of smoking per day 1-10 ________ 11-20________ 21-30________ 31-40________

41-50________ 51-60________ >60________

File number: ____________

1.3 Passive smoking

1.3.1 Years of Passive Smoking ________year

2. History of alcohol drinking

2.1 Alcohol drinking Yes. ___________ No. ___________

2.2 The amount of alcohol consuming per day ___________

3. Personal habits

3.1 Raw vegetable Never___________ Often___________ Everyday___________

3.2 Fried food Never___________ Often___________ Everyday___________

3.3 Spicy food Never___________ Often___________ Everyday___________

3.4 Pickled food Never___________ Often___________ Everyday___________

3.5 Bacon Never___________ Often___________ Everyday___________

3.6 Raw Garlic Never___________ Often___________ Everyday___________

3.7 Drinking unboiled water Never___________ Often___________ Everyday___________

3.8 Hand washing Never___________ Often___________ Everyday___________

3.9 Stay up Never___________ Often___________ Everyday___________

3.10 Washing fruit Never___________ Often___________ Everyday___________

3.11 Peeling fruit Never___________ Often___________ Everyday___________

3.12 Source of drinking water Piped water___________ barreled water___________

4. Family Habits

4.1 Raising domestic animals Never___________ Often___________ Everyday___________

4.2 Shared tableware Never___________ Often___________ Everyday___________

4.3 Shared cups Never___________ Often___________ Everyday___________

4.4 Shared tooth-brushing cups Never___________ Often___________ Everyday___________

4.5 Washing tableware with cleaner Never_________ Often___________ Everyday___________

4.6 Eating by individual serving Never___________ Often___________ Everyday___________

5. Digestive disease

5.1 Chronic gastritis No___________ Once___________ Yes___________

5.2 Gastric ulcer No___________ Once___________ Yes___________

5.3 Duodenal ulcer No___________ Once___________ Yes___________

5.4 Cholelithiasis No___________ Once___________ Yes___________

5.5 Family history of gastrointestinal cancer No___________ Once___________ Yes___________

5.6 Gastric disease of spouse No___________ Once___________ Yes___________

5.7 History of gastrointestinal endoscopy No___________ Once___________ Yes___________

5.8 Other digestive diseases ___________

6. Other diseases

6.1 Pulmonary diseases No___________ Once___________ Yes___________

Name of disease___________

6.2 Autoimmune diseases. No___________ Once___________ Yes___________

File number: ____________

Name of disease___________

6.3 Interval from antibiotics use to Hp detection <1 week___________ 1-2 weeks___________

2-4 weeks ___________ >4 weeks ___________

6.4 Interval from NSAIDs use to Hp detection <1 week___________ 2-3 weeks___________ 4 weeks-3 months ___________ >3 months ___________

7. Knowledge about Hp

7.1 Purpose of ^{13}C-UBT Unclear ___________ intermediate ___________clear ___________

7.2 What is Hp Unclear___________ intermediate ___________clear ___________

7.3 Route of transmission Unclear___________ intermediate ___________clear ___________

7.4 Hp-related diseases Unclear___________ intermediate ___________clear ___________

7.5 Interval of reexamination Unclear___________ intermediate ___________clear ___________

7.6 History of Hp detection Unclear___________ intermediate ___________clear ___________

7.7 Hp infection in family Unclear___________ intermediate ___________clear ___________

中石油冀东油田员工
健康随访表

档 案 编 号 □□□□□□□□

体 检 编 号 □□□□□□□□□□

体 检 人 姓 名

体 检 人 单 位

体 检 日 期 □□□□年□□月□□日

调 查 者 姓 名 □□

冀东油田卫生服务中心编印
2015

填 写 说 明

1. 填写记录请使用蓝黑色钢笔或签字笔清楚填写。

2. 体检表填写务必准确、清晰，不得随意涂改，错误之处纠正时需用红色钢笔或签字笔在错误处居中划一道横线，并签署修改者姓名及修改时间，原改处应能清晰可见。不要掩盖填入的原始数据，不要用橡皮擦、用改正液遮盖或划许多道线。

3. 举例：~~3.24~~ 8.2 李某某 07/04/28

4. 根据职工本人的具体情况，按要求填写登记表的每一项。在“□”内划“×”表示选择此项。在“_____”内填写有关的文字说明，要求字迹工整，应用规范的医学术语，不得应用简写或英文缩写。

5. 缺失或不可得的信息，请在相应的横线上面填写“NA”。

6. 所有表格上的日期都以“年／月／日”的形式表示，包括职工本人的出生日期。如果不知道具体日期，请用“NA”表示，以“年／月／NA”的形式填入日期，请尽可能填入完整的阳历日期。所有时间均采用 24 小时计时制，如下午 5 点应填写为“17:00”。

档案编号 [| | | | | | |]

知情同意书

背景：我们邀请您参加中石油冀东油田员工健康随访研究。本研究旨在了解冀东油田职工的健康状况及疾病的患病情况，并随访员工心脑血管等系统疾病的发生和发展，以期制定相应的积极、有效地预防和治疗措施，提高员工的健康水平和生活质量。

操作步骤：若您同意参与此项研究，您需协助医生完成调查表中所需填写项目，内容涉及您的基本信息、生活状况及疾病情况（包括发病时间、诊治情况等），您还需要留下可靠的联系方式（包括电话和住址），以便医生及时将您的健康信息反馈给您及进行随访。

获益：您可以全面了解您的健康情况，并获得疾病的预防信息。

风险：参与该项研究您不会有任何风险。

保密性：所有信息均保密，您的姓名、电话和联系方式及其他基本信息不会泄漏给其他非研究人员和组织。

自愿性：您参加本研究完全出于自愿，拒绝参加将不会影响您本次的体检。

知情同意声明

我已经阅读了上述信息，所有的研究规程均已解释清楚，我已经完全理解。同时我获得了讨论和问问题的机会并且所有问题都已获得了满意的答复。

因此，我同意参加此项研究。

体检者或家属签字：____________

日期：[| | |] 年 [|] 月 [|] 日

档案编号 □□□□□□□□

第一部分 基本信息

A1、姓名：________　　A2、性别：男 □　女 □　　A3、颈围：□□ cm

A4、联系电话（移动）：□□□□□□□□□□□

A5、联系电话（固定）：□□□□ - □□□□□□□

A6、身份证号：□□□□□□□□□□□□□□□□□□□

A7、家庭住址：________省______市________县（区）____________乡（镇）

A8、人均住房面积（平方米）：*小于等于 30* □　*30 到 60* □　*大于等于 60* □

A9、家庭常驻人口数（人）：*1 人* □　*2-4 人* □　*大于等于 5 人* □

A10、家庭周围环境：*好* □　*较好* □　*差* □

A11、家庭卫生条件：□　□　□

A5、您的文化程度：*未接受过正规学校教育* □　*小学未毕业* □　*小学* □　*初中* □　*高中／中专／技校* □　*大专* □　*本科* □　*研究生及以上* □

A6、您目前的婚姻状态：*未婚* □　*已婚* □　*离婚* □　*分居* □　*丧偶* □　*其他* □

第二部分 个人健康信息

1. 高血压病史

1.1 自上次体检（2013 年到 2014 年）至今，是否被医生诊断为高血压：*是* □　*否* □ → ***跳转到 2***

1.2 既往血压最高：*140/90mmHg 至 160/100mmHg* □　*160/100mmHg 至 180/110mmHg* □　*大于 180/110mmHg* □

1.3 首次诊断时间：□□□□ 年 □□ 月 □□ 日

1.4 目前是否使用降压药：*是* □　*否* □

档案编号 □□□□□□□□

1.5 目前正在服用的药物 (多选):

利尿剂	β受体阻滞剂	钙拮抗剂	血管紧张素转换酶抑制剂	血管紧张素 II 受体拮抗剂
□	□	□	□	□

叶酸制剂	α受体阻滞剂	复合制剂	中药制剂及其他
□	□	□	□

2. 糖尿病史

2.1 自上次体检（2013 年到 2014 年）至今，是否被医生诊断为糖尿病： 是 □ 否 □ → **跳转到 3**

2.2 首次诊断时间： □□□□ 年 □□ 月 □□ 日

2.3 糖尿病类型： I 型 □ II 型 □ 不详 □

2.4 目前是否使用降糖药： 是 □ 否 □

2.5 目前正在使用的药物 (多选):

磺脲类	双胍类	葡萄糖苷酶抑制剂	噻唑烷二酮类	非胰岛素促分泌剂	胰岛素制剂	中药制剂及其他
□	□	□	□	□	□	□

3. 高脂血症病史

3.1 自上次体检（2013 年到 2014 年）至今，是否被医生诊断为高脂血症： 是 □ 否 □ → **跳转到 4**

3.2 首次诊断时间： □□□□ 年 □□ 月 □□ 日

3.3 目前是否使用降脂药： 是 □ 否 □

3.4 调脂药物种类 (多选):

他汀类	贝特类	胆酸螯合树脂类	烟酸及其衍生物	中药制剂及其他
□	□	□	□	□

3.5 服用降脂药过程中是否有不良反应： 是 □ 否 □ → **跳转到 4**

3.6 不良反应种类 (多选):

肌肉酸痛	肝功能异常	腹胀、恶心等消化道不适	其他
□	□	□	□

4. 心血管病史

4.1 自上次体检（2013 年到 2014 年）至今，是否被医生诊断为心血管疾病（首次）： 是 □ 否 □ → **跳转到 5**

档案编号 □□□□□□□□

请选择类型(多选):　诊断时间　诊断医院

□ 房颤/房扑	□□□□年□□月□□日	________
□ 心力衰竭	□□□□年□□月□□日	________
□ 心肌梗死	□□□□年□□月□□日	________
□ 冠心病	□□□□年□□月□□日	________
□ 外周动脉粥样硬化	□□□□年□□月□□日	________
	**手术时间**	**手术医院**
□ 心脏置入支架	□□□□年□□月□□日	________

5. 脑血管病史

5.1 自上次体检(2013年到2014年)至今,是否被医生诊断为脑血管疾病(首次): *是* □ *否* □ → *跳转到6*

请选择类型(多选):　诊断时间　诊断医院

□ 缺血性卒中	□□□□年□□月□□日	________
□ 脑出血	□□□□年□□月□□日	________
□ 蛛网膜下腔出血	□□□□年□□月□□日	________
□ 短暂性脑缺血发作	□□□□年□□月□□日	________
□ 其他	□□□□年□□月□□日	________

6. 肿瘤病史

6.1 自上次体检(2013年到2014年)至今,是否被医生诊断为肿瘤(首次): *是* □ *否* □

请选择类型(多选):　诊断时间　诊断医院

□ 乳腺癌	□□□□年□□月□□日	________
□ 肺癌	□□□□年□□月□□日	________
□ 胃癌	□□□□年□□月□□日	________
□ 肠癌	□□□□年□□月□□日	________

档案编号 □□□□□□□□

	诊断时间	诊断医院
□ 胰腺癌	□□□□年□□月□□日	______
□ 肾癌	□□□□年□□月□□日	______
□ 肾盂或输尿管癌	□□□□年□□月□□日	______
□ 膀胱癌	□□□□年□□月□□日	______
□ 前列腺癌	□□□□年□□月□□日	______
□ 甲状腺癌	□□□□年□□月□□日	______
□ 淋巴瘤	□□□□年□□月□□日	______
□ 白血病	□□□□年□□月□□日	______
□ 宫颈癌	□□□□年□□月□□日	______
□ 子宫内膜癌	□□□□年□□月□□日	______
□ 其他	□□□□年□□月□□日	______

第三部分 饮食、生活习惯

1. 吸烟史

1.1 是否吸烟：*现在吸* □ *从不吸* □ *曾经吸，现已戒烟 12 个月* □ *曾经吸，现已戒烟 <12 个月* □

1.2（若现在吸烟）每日平均吸烟支数：

1-10 支 □ *11-20 支* □ *21-30 支* □ *31-40 支* □ *41-50 支* □ *51-60 支* □ *61 支及以上* □

1.3 是否被动吸烟（家庭或工作环境中经常有人吸烟）：*有* □ *无* □

1.3.1 如有，被动吸烟的年限 □□ 年

2. 饮酒史

2.1 饮酒频率：*不饮酒* □ *<1 个标准饮酒量／* □ *<2 个标准饮酒量／日* □ *2-4 个标准饮酒量／日* □ *≥ 5 个标准饮酒量／日* □ *饮酒但剂量不详* □ *已戒酒* □ *不详* □

（1 个标准饮酒量相当于 120ml［2.5 两］葡萄酒、360ml［1 听］啤酒或 45ml［1 两左右］白酒）

档案编号 □□□□□□□□

3. 个人生活习惯

	无或偶然	经常	每天
3.1 生食蔬菜：	□	□	□
3.2 进食油炸食品：	□	□	□
3.3 进食辛辣食品：	□	□	□
3.4 进食腌制食品：	□	□	□
3.5 进食熏肉、腊肉等：	□	□	□
3.6 进食生大蒜：	□	□	□
3.7 饮生水（未煮开的水）:	□	□	□
3.8 饭前便后洗手：	□	□	□
3.9 熬夜：	□	□	□
3.10 蔬菜水果清洗：	□	□	□
3.11 水果是否削皮：	□	□	□
3.12 经常饮用水源：	□（水龙头水）	□（桶装水）	□（市售瓶装水）

4. 家庭生活习惯

	有	*无*
4.1 室内养动物：	□	□

	无或偶然	经常	每天
4.2 共用餐具：	□	□	□
4.3 共用水杯：	□	□	□
4.4 共用涮牙杯：	□	□	□

4.5 碗筷清洗：	*用洗洁精* □	*多半用洗洁精* □	*较少用洗洁精* □	*从不用洗洁精* □
4.6 分餐或公筷：	*无或偶然* □	*有时* □	*经常* □	*每天* □

5. 消化道疾病

	无	曾有	现有
5.1 慢性胃炎病史：	□	□	□
5.2 胃溃疡病史：	□	□	□
5.3 十二指肠溃疡病史：	□	□	□

档案编号 ☐☐☐☐☐☐☐☐

5.4 慢性胆囊炎胆结石病史: 无 ☐ 曾有 ☐ 现有 ☐

取石或手术 否 ☐ 是 ☐

5.5 胃肠道肿瘤家族史: 无 ☐ 曾有 ☐ 现有 ☐

手术干预 否 ☐ 是 ☐

5.6 配偶胃病史: 无 ☐ 曾有 ☐ 现有 ☐

5.7 胃镜检查史: 无 ☐ 曾有 ☐ 现有 ☐ 次数: ☐☐

5.8 其他消化道病史(如肝硬化): 无 ☐ 曾有 ☐ 现有 ☐ 疾病名称:________

6. 其他疾病

6.1 呼吸道病史(如肺结核、支气管扩张、慢阻肺)

无 ☐ 曾有 ☐ 现有 ☐ 疾病名称:________

6.2 自身免疫疾病史: 无 ☐ 有 ☐ (如有,以下多选)

痛风 ☐ 类风湿性关节 ☐ 其他关节炎 ☐ 系统性红斑狼 ☐ 过敏性紫癜 ☐ 银屑病(或牛癣) ☐ 强直性脊柱炎 ☐

6.3 服用抗生素时限(距 HP 检测时间): 少于 1 周 ☐ 1-2 周 ☐ 2-4 周 ☐ 4 周及以上 ☐

6.4 服用非甾体抗炎药时限(距 HP 检测时间)
(阿司匹林、吲哚美辛、扑热息痛、布洛芬、贝诺酯、芬必得、吡罗昔康等)

少于 1 周 ☐ 2-3 周 ☐ 4 周 -3 月 ☐ 3 月及以上 ☐

7. 幽门螺杆菌(HP)知晓状况

	不了解	略有了解	了解
7.1 这次吹气检测的目的	☐	☐	☐
7.2HP 是什么	☐	☐	☐
7.3HP 的传播途径	☐	☐	☐
7.4HP 与哪些疾病相关	☐	☐	☐
7.5HP 感染的治疗用药	☐	☐	☐

档案编号 □□□□□□□□

7.6HP 感染的复查时间　*不了解* □　*略有了解* □　*了解* □

7.7 自己及家人是否检测过　*无* □　*单人单次* □　*单人多次* □　最高次数：□□

7.8 家族中是否存在 HP 感染　*否* □　*是* □　*不详* □

8 药物使用情况（您**用过或正在使用**以下哪些**药物**？**若平均每月不到 1 次不用填写**）(多选)

药物	是　否	开始使用时间	平均使用天数			
			每月 **1-3** 天	每周 **1-2** 天	每周 **3-4** 天	每周 **5-7** 天
阿司匹林	□　□	□□□□年□□月	□	□	□	□
止痛药	□　□	□□□□年□□月	□	□	□	□
他汀类	□　□	□□□□年□□月	□	□	□	□
中药	□　□	□□□□年□□月	□	□	□	□

9. 睡眠状况

9.1 在过去的 2 年里，您是否使用药物改善睡眠（如安眠药等）：

从未使用 □　*每月<1 次* □　*每月 1-3 次* □　*每周 1 次* □　*每周 2-6 次* □　*每天* □

9.2 睡眠打鼾么？　*从不* □　*每周<1 次* □　*每周 1-5 次* □　*几乎每天* □　*不知道* □

9.3 鼾声比平时说话声还响或关着门也能听到吗？　*否* □　*是* □　*不知道* □

9.4 有人说你睡觉时有呼吸暂停吗？（>10 秒）　*否* □　*是* □　*不知道* □

9.5 是否曾经被告知自己在做噩梦的时候，会随着梦境动手脚？

否 □　*是，但只有 1-2 次* □　*是，至少三次以上* □

档案编号 ☐☐☐☐☐☐☐☐

第四部分 请问你的头型最接近以下哪个

男性

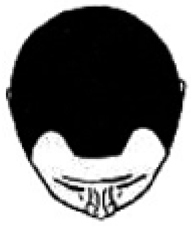	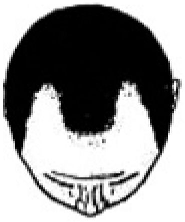	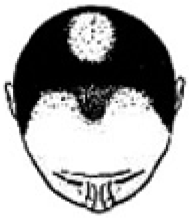	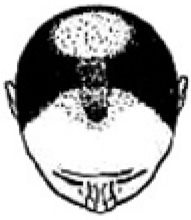	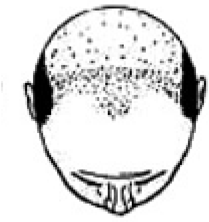
☐	☐	☐	☐	☐

女性

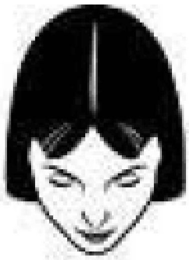	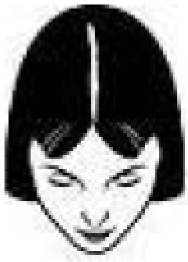	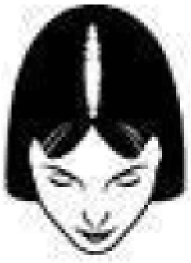	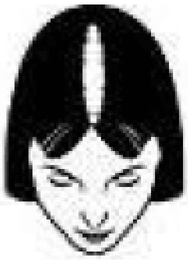	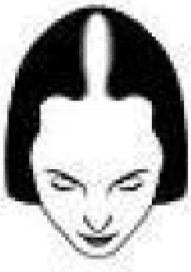
☐	☐	☐	☐	☐
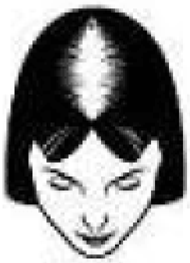	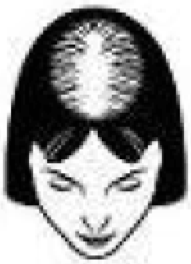	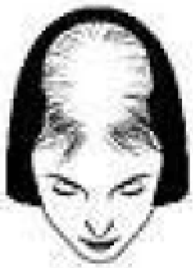	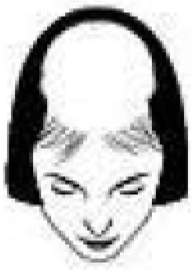	
☐	☐	☐	☐	

第五部分 亚健康状况调查问卷

请您就**最近三个月**的情况，回答下列问题

B1 您觉得疲劳吗（在运动量没有明显增加的情况下）？

完全没有	*很少有*	*有*	*多数有*	*几乎总是*
☐	☐	☐	☐	☐

档案编号 □□□□□□□□

B2 您的疲劳在休息后能缓解吗？

完全缓解 □　*明显缓解* □　*有所缓解* □　*略有缓解* □　*几乎不能缓解* □

B3 在工作时您有困倦或倦怠的现象吗

完全没有 □　*很少有* □　*有* □　*多数有* □　*几乎总是* □

B4-B24 对于下表中所列的问题，在过去的三个月内，这些症状在您身上发生的频率是怎样的？最大部分时候有、有时候有、很少或是从来没有？（每行单选）

症状	完全没有	很少有	有	多数有	几乎总是
B4 感觉头痛	□	□	□	□	□
B5 感觉头晕	□	□	□	□	□
B6 眼睛酸胀	□	□	□	□	□
B7 咽喉疼痛	□	□	□	□	□
B8 肌肉和关节经常感到发僵或僵硬	□	□	□	□	□
B9 肩、颈或腰部酸痛	□	□	□	□	□
B10 走路时感到双腿沉重	□	□	□	□	□
B11 静息时感到气短	□	□	□	□	□
B12 胸闷	□	□	□	□	□
B13 心悸	□	□	□	□	□
B14 食欲减退	□	□	□	□	□
B15 胃部不适	□	□	□	□	□
B16 消化不良	□	□	□	□	□
B17 低热或怕冷	□	□	□	□	□
B18 入睡有困难	□	□	□	□	□
B19 多梦或易被惊醒的情形	□	□	□	□	□
B20 记忆力减退	□	□	□	□	□
B21 反应能力下降	□	□	□	□	□
B22 注意力不集中	□	□	□	□	□
B23 没有原因的心烦意乱	□	□	□	□	□
B24 紧张、焦虑现象	□	□	□	□	□

档案编号 □□□□□□□□

B25 在最近一年中，您是否患过感冒？

完全没有	*很少有*	*有*	*多数有*	*几乎总是*
☐	☐	☐	☐	☐

第六部分 认知功能调查表

Mini-Mental State Examination（MMSE）简易精神状态检查

项目	不正确	正确
**定向力**		
1. 今天是这个月的几号？	☐	☐
2. 今年是哪一年？	☐	☐
3. 现在是几月份？	☐	☐
4. 今天是星期几？	☐	☐
5. 现在是什么季节？	☐	☐
6. 这家医院的名字是什么？	☐	☐
7. 我们现在是在第几层楼？	☐	☐
8. 我们现在在哪个城市 （城镇）？	☐	☐
9. 我们现在在哪个省 （或州、或地区、或县、或岛屿）？	☐	☐
10. 我们现在在哪个国家？	☐	☐
**即刻记忆**		
**现在我要说三样东西的名称，在我讲完之后，请您重复说一遍，请您记住这三样东西，因为等一下要再问您的：（以第一次回答记分）**		
11. 回答出 “ 皮球 ”	☐	☐
12. 回答出 “ 国旗 ”	☐	☐
13. 回答出 “ 树木 ”	☐	☐
**注意力与计算能力**		
**100 减 7，连续减 5 次** （若错了，但下一个回答是对的，得 **1** 分）		
14. 回答出 “93”	☐	☐
15. 回答出 “86”	☐	☐
16. 回答出 “79”	☐	☐

档案编号 □□□□□□□□

项目	不正确	正确
17. 回答出 “72”	☐	☐
18. 回答出 “65”	☐	☐
**回忆能力**		
**现在请您告诉我，刚才我要您记住的三样东西是什么？**		
19. 回答出 “ 皮球 ”	☐	☐
20. 回答出 “ 国旗 ”	☐	☐
21. 回答出 “ 树木 ”	☐	☐
**语言能力**		
**访问员拿出手表，并问 “ 请问这是什么？ ” 然后再拿出一支铅笔，并询问同样问题**		
22. 回答出 “ 手表 ”	☐	☐
23. 回答出 “ 铅笔 ”	☐	☐
**重复**		
24. 请您跟我说：“ 四十四只石狮子 ”	☐	☐
**三步命令：给患者一张白纸并告诉他 / 她， “ 用您的右手拿起纸，对折并把纸放在地板上 ”**		
25. 患者用右手拿起纸？	☐	☐
26. 患者将纸对折？	☐	☐
27. 患者把纸放在地板上？	☐	☐
**阅读：访问员把写有 “ 闭上您的眼睛 ” 大字的卡片交给患者，请患者照着这张卡片所写的去做**		
28. 患者闭上了眼睛？	☐	☐
**书写：请患者写一句完整的、有意义的句子（必须有主语、动词，并有意义）记下句子**		
29. 患者写了一个句子？	☐	☐

档案编号 □□□□□□□□

复制		
30. 请您按样子画图	□	□

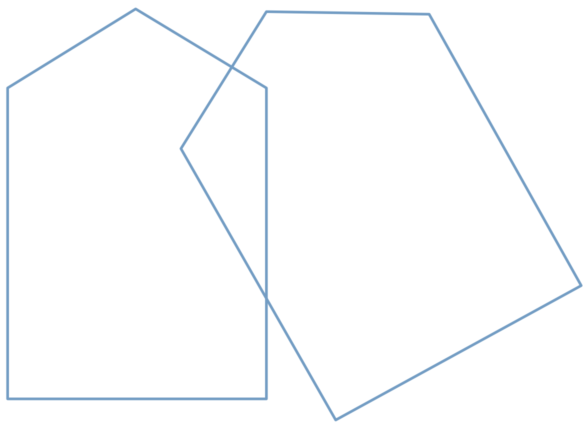

文化程度:

文盲	小学	中学程度（包括中专）	大学程度（包括大专）
□	□	□	□

备注: 文盲: 未受教育
小学: 受教育年限≤6 年
中学以上: 受教育年限＞6 年

得分：□□ 分
